# C-reactive Protein-Albumin-Lymphocyte (CALLY) Index in Patients With Sepsis: An Observational Study

**DOI:** 10.7759/cureus.103423

**Published:** 2026-02-11

**Authors:** Shubhransu Patro, Sidharth S Pattnaik, Parmarth Arora, Arushi Choudhary, Vibha Sharma, Sai Sri Karlapudi, Varun Jindal

**Affiliations:** 1 General Medicine, Kalinga Institute of Medical Sciences, Bhubaneswar, IND

**Keywords:** cally index, correlation analysis, c-reactive protein (crp), infection control measures, lymphocyte count, multi organ dysfunction syndrome (mods), sequential organ failure assessment (sofa), serum albumin, severe sepsis, vasopressor

## Abstract

Background and objectives: Sepsis and multiorgan dysfunction syndrome (MODS) are serious global health concerns. Patients with critical illnesses are assessed using the sequential organ failure assessment (SOFA) score for morbidity. The C-reactive protein (CRP)-albumin-lymphocyte (CALLY) index is a novel composite biomarker for sepsis. It is composed of three parameters: absolute lymphocyte count, CRP, and serum albumin. The purpose of this study was to examine the CALLY index in male and female sepsis patients. Additionally, we correlated the SOFA score and CALLY index of the study participants at admission.

Methods: This observational study was carried out from August 2025 to December 2025 at Kalinga Institute of Medical Sciences (KIMS), Bhubaneswar, India. Adult patients of both sexes fulfilling Sepsis-3 criteria were included in this study. We recorded their absolute lymphocyte count, CRP, and serum albumin for the computation of the CALLY index. SOFA scores were noted at days 1, 3, and 7. The categorical and continuous variables were assessed with the Chi-squared and Wilcoxon tests, respectively. We used the Spearman correlation to assess the association between the SOFA score at day 1 and the CALLY index for the subjects. R software (version 4.3.2; R Foundation for Statistical Computing, Vienna, Austria) was used for data analysis.

Results: Two hundred sixty-three patients were analyzed in this study. Their median age was 62.0 (52.0-73.0) years. One hundred sixty-one (61.22%) subjects were males. The median hospitalization duration was 12.0 (9.0-16.0) days. Two hundred fourteen (81.37%) subjects had MODS. The median CRP, serum albumin, and lymphocyte count values of the study population were 112.97 (77.95-206.82) mg/L, 2.78 (2.36-3.17) g/dL, and 1.09 (0.67-1.72) x 10^9^/L. The median CALLY index of the subjects was 2.48 (1.34-4.94). The median SOFA score during admission was 5.0 (3.0-8.0). There was a negative correlation between SOFA score at day 1 and CALLY index (-0.299, 95% confidence interval (CI): -0.406 to -0.185, p < 0.001). Male and female subjects also showed similar negative correlations.

Conclusion: Male and female subjects with sepsis had comparable CRP, serum albumin, lymphocyte count, CALLY indices, and SOFA scores. The CALLY index and SOFA score were negatively correlated. Our study findings cannot be generalized due to a small sample size and missing data on antibiotics used; hemodynamic, renal, hepatic, and glycemic parameters; comorbidities; concomitant medications; daily fluid intake; and urine output.

## Introduction

Dysregulated host immune response leading to multiorgan dysfunction syndrome (MODS) is the hallmark of sepsis. It increases the need for intensive care units (ICUs), assisted ventilation, and vasopressors. Sepsis remains a significant global health concern [1-4]. The outcome of sepsis is dependent on promptly identifying subjects who are at risk for rapid clinical deterioration. However, the standard approaches are often insufficient or difficult to apply in the early stages of care [5,6].

The sequential organ failure assessment (SOFA) score was designed to assess the seriousness of illness in critically ill patients [7]. However, organ failure is not an event, but a process. Therefore, it should be viewed as a spectrum that can be measured objectively. To simplify computation, there should be fewer variables that are easily, quickly, and consistently available across institutions [8,9]. Procalcitonin, C-reactive protein (CRP), adrenomedullin, interleukin-6 (IL-6), and intercellular adhesion molecule-1 (ICAM-1) are the most commonly evaluated biomarkers for sepsis [4,10,11]. Higher cost and limited availability render these biomarkers as less feasible alternatives for quantifying sepsis progression [3,4,12].

The CRP-albumin-lymphocyte (CALLY) index is a new composite biomarker for sepsis. It consists of three parameters, i.e., serum albumin (nutritional-metabolic reserve), CRP (inflammation), and absolute lymphocyte count (immune competence). The CALLY index is computed as ((Albumin in g/L) * (Lymphocyte count in 1,000 cells/µL))/(CRP in mg/dL) [13-16]. The CALLY index has shown promise across a spectrum of acute and chronic inflammatory states, including cardiorenal syndrome and acute ischemic stroke [13,15]. Lower CALLY index values at admission may be associated with an elevated risk of organ dysfunction, hemodynamic instability, and ICU admission [14,16]. The CALLY index demonstrated an inverse relationship with SOFA scores in critically ill sepsis patients [15]. Calculating and monitoring the CALLY index at healthcare institutions seems quick and inexpensive. Hence, we conducted this study to evaluate the CALLY index of male and female patients with sepsis. We also correlated the study subjects' CALLY index and SOFA score during admission.

## Materials and methods

We conducted this observational study from August 2025 to December 2025 at the Kalinga Institute of Medical Sciences (KIMS) in Bhubaneswar, India. The Institutional Ethics Committee granted ethics approval for this study (KIIT/KIMS/IEC/2269/2025, dated July 27, 2025).

Study criteria

We included adult patients of both sexes admitted to our hospital during the study period who fulfilled the Sepsis-3 criteria [17]. We excluded the patients with any malignancy, immunocompromised state, diabetes mellitus, ongoing chemotherapy, steroids, or blood component transfusion. We also excluded the patients referred from other hospitals and those who died within or stayed for less than seven days.

Study procedure

We recorded the sociodemographic data (i.e., age, sex, socioeconomic status, and marital status) from the subjects' discharge summaries. We used the Kuppuswamy classification to classify the socioeconomic class [18]. We noted the following clinical parameters upon admission to the hospital: CRP, serum albumin, and absolute lymphocyte count. The SOFA scores were assessed on the day of admission, day 3, and day 7, as per the SOFA scoring system [7]. We computed the CALLY index from the collected values for CRP, serum albumin, and absolute lymphocyte count. The CALLY index is computed as ((Albumin in g/L) * (Lymphocyte count in 1,000 cells/µL))/(CRP in mg/dL) [13-16]. The normal ranges of CRP, albumin, and lymphocyte count are <1 mg/L, 3.5-5.0 g/dL, and 1.0-4.8 x 10^9^ cells/L, respectively. We assessed the correlation between the baseline SOFA score and the CALLY index for the subjects. We also performed the subgroup analysis of the correlation based on difference in SOFA score at day 7 (<2 or ≥2), outcome (i.e., death or discharge), MODS (present or not), vasopressor (required or not), duration of hospitalization (>14 or ≤14 days), and age group (≤65 or >65 years).

Statistical analysis

For this study, we used a non-probability consecutive sampling. The continuous variables were reported as medians and interquartile ranges (IQRs). Categorical variables were reported as numbers and percentages. The categorical and continuous variables were analyzed with the Chi-squared test and the Wilcoxon test, respectively. We used the Spearman correlation to assess the association between baseline SOFA scores and CALLY indices in the subjects. We reported the correlation coefficients with 95% confidence intervals (CIs). For data analysis, we used version 4.3.2 of the R software (R Foundation for Statistical Computing, Vienna, Austria) [19]. A p-value ≤ 0.05 was considered statistically significant.

## Results

During the study period, 2,194 patients were admitted to the medicine ward. One thousand two hundred ninety patients had diabetes. Three hundred sixty-two patients either died or left the hospital against medical advice within seven days of their admission. Two hundred seventy-nine did not develop sepsis. The data from the remaining 263 patients were analyzed in this study. Table 1 shows the clinical and sociodemographic data of the 263 patients. The median age of the study population was 62.0 (52.0-73.0) years. One hundred sixty-one (61.22%) male subjects were included in the study population. The median hospitalization duration was 12.0 (9.0-16.0) days. Eighty-one (30.80%) subjects were hospitalized for >14 days. Two hundred fourteen (81.37%) subjects had MODS. Vasopressors were required for 141 (53.61%) patients. The median SOFA score during admission was 5.0 (3.0-8.0).

**Table 1:** Demographic and clinical parameters of the study participants The continuous variables were reported as medians and IQRs. Categorical variables were reported as counts and percentages. The normal ranges of CRP, albumin, and lymphocyte count are <1 mg/L, 3.5-5.0 g/dL, and 1.0-4.8 x 10^9^ cells/L, respectively. The categorical and continuous variables are assessed with the Chi-squared and Wilcoxon tests, respectively. Statistical significance is set at p < 0.05. The test statistics for the categorical and continuous variables were the Chi-squared value and the t-value, respectively. IQR: interquartile range; CRP: C-reactive protein; CALLY index: CRP-albumin-lymphocyte index; SOFA: sequential organ failure assessment; MODS: multiorgan dysfunction syndrome.

Parameters	Total (n = 263)	Male (n = 161)	Female (n = 102)	Statistical test used	Test statistics	p-value
Age (years)	62.00 (52.00-73.00)	65.00 (53.00-75.00)	60.00 (50.00-71.75)	Wilcoxon test	63.532	<0.001
Elderly (age > 65 years)	105 (39.92%)	79 (49.07%)	26 (25.49%)	Chi-squared test	84.871	<0.001
Marital status						
Married	221 (84.03%)	133 (82.61%)	88 (86.27%)	Chi-squared test	93.038	<0.001
Unmarried	23 (8.75%)	17 (10.56%)	6 (5.88%)			
Divorced/widowed	19 (7.22%)	11 (6.83%)	8 (7.84%)			
Socioeconomic status						
Low	184 (69.96%)	121 (75.16%)	63 (61.76%)	Chi-squared test	91.773	<0.001
Lower middle	67 (25.48%)	32 (19.88%)	35 (34.31%)			
Upper middle	12 (4.56%)	8 (4.96%)	4 (3.92%)			
CRP (mg/L)	112.97 (77.95-206.82)	111.88 (79.17-198.71)	117.20 (75.20-212.26)	Wilcoxon test	0.261	0.970
Serum albumin (g/dL)	2.78 (2.36-3.17)	2.81 (2.40-3.24)	2.65 (2.30-3.11)	Wilcoxon test	1.645	0.111
Absolute lymphocyte count (10^9^/L)	1.09 (0.67-1.72)	1.06 (0.67-1.61)	1.18 (0.68-1.92)	Wilcoxon test	1.590	0.200
CALLY index	2.48 (1.34-4.94)	2.43 (1.39-4.45)	2.57 (1.26-5.31)	Wilcoxon test	0.719	0.612
Hospitalization (days)	12.0 (9.0-16.0)	12.0 (9.0-17.0)	12.0 (9.0-14.8)	Wilcoxon test	0.731	0.673
SOFA score at day 1	5.0 (3.0-8.0)	5.0 (3.0-8.0)	4.0 (2.0-7.0)	Wilcoxon test	1.603	0.134
SOFA score at day 3	6.0 (2.0-9.0)	6.0 (3.0-9.0)	5.0 (2.0-9.0)	Wilcoxon test	1.628	0.117
SOFA score at day 7	5.0 (2.0-9.0)	6.0 (2.0-9.0)	4.0 (2.0-9.0)	Wilcoxon test	2.703	0.087
Difference in SOFA scores	0.0 (-2.0-2.0)	0.0 (-2.0-2.0)	0.0 (-2.0-2.0)	Wilcoxon test	0.515	0.869
MODS	214 (81.37%)	136 (84.47%)	78 (76.47%)	Chi-squared test	3.804	0.017
Vasopressor required	141 (53.61%)	85 (52.80%)	56 (54.90%)	Chi-squared test	4.023	0.008
Long duration of stay (hospitalization > 14 days)	81 (30.80%)	55 (34.16%)	26 (25.49%)	Chi-squared test	32.007	<0.001

**Figure 1:**
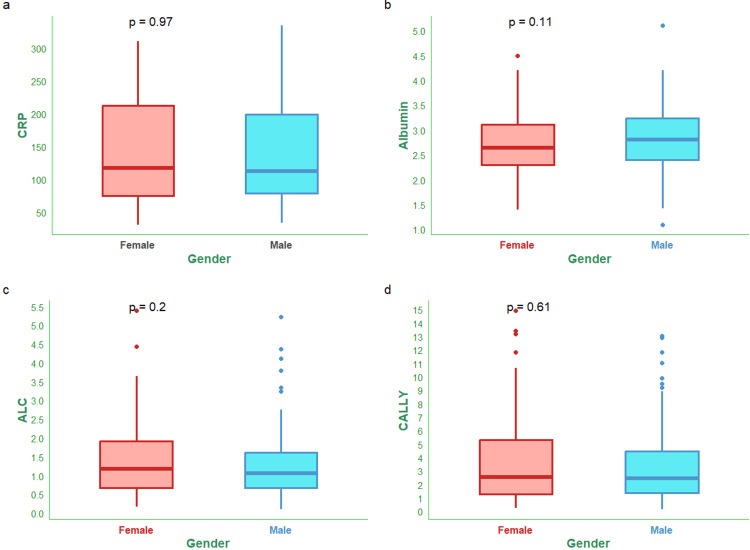
CALLY index and its components of the female and male participants The box-and-whisker plots show the CALLY index and its components (i.e., CRP, albumin, and lymphocyte count) of female and male participants. Panels (a-d) show the CRP (mg/L), serum albumin (g/dL), absolute lymphocyte count (x10^9^/L), and CALLY index, respectively. Statistical significance is set at p < 0.05. The Wilcoxon test was used for the between-group comparisons. CRP: C-reactive protein; ALC: absolute lymphocyte count; CALLY index: CRP-albumin-lymphocyte index.

Figure 1 shows the CALLY index and its components (i.e., CRP, serum albumin, and lymphocyte count) of male and female participants. The median CRP values of male and female subjects were 111.88 (79.17-198.71) mg/L and 117.20 (75.20-212.26) mg/L (Figure 1a). The median serum albumin values of male and female subjects were 2.81 (2.40-3.24) g/dL and 2.65 (2.30-3.11) g/dL (Figure 1b). The median absolute lymphocyte counts of male and female subjects were 1.06 (0.67-1.61) x 10^9^/L and 1.18 (0.68-1.92) x 10^9^/L (Figure 1c). The median CALLY indices for male and female subjects were 2.43 (1.39-4.45) and 2.57 (1.26-5.31), respectively (Figure 1d).

**Figure 2:**
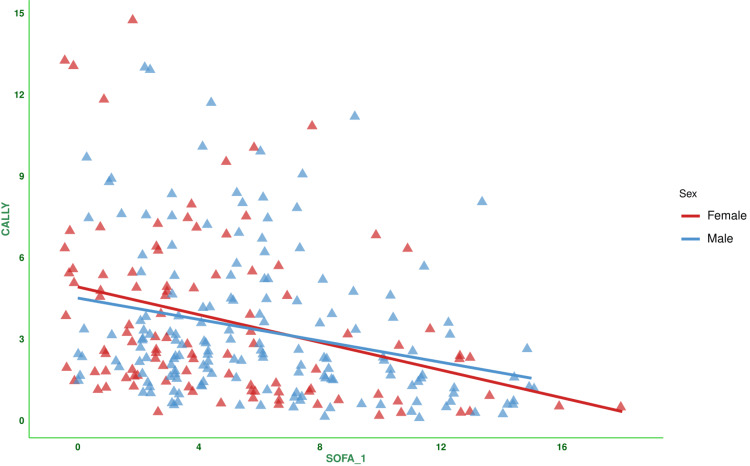
Correlation between the CALLY index and the SOFA score of the study population The jitter plots show the correlation between the baseline SOFA score and the CALLY index in the study population. The Spearman correlation was used to check the association. Statistical significance is set at p < 0.05. CRP: C-reactive protein; CALLY index: CRP-albumin-lymphocyte index; SOFA: sequential organ failure assessment; SOFA_1: SOFA score at day 1.

**Figure 3:**
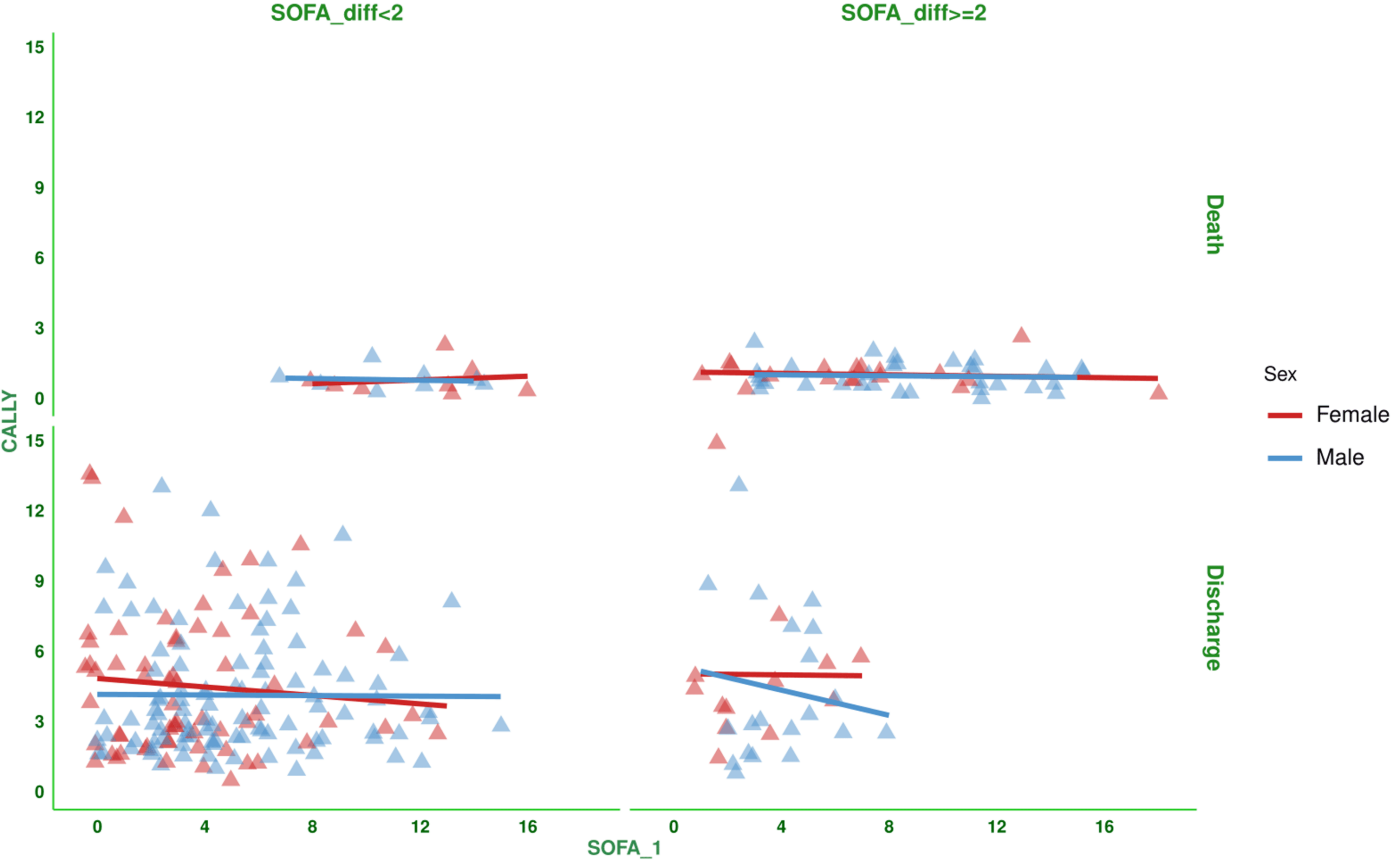
Correlation between the CALLY index and the SOFA score based on the difference in SOFA scores and the outcome of the study population The jitter plots show the correlation between the baseline SOFA score and the CALLY index in the study population. The vertical and horizontal grids denote differences in the SOFA score at day 7 from that at day 1 (<2 or ≥2) and outcome (i.e., death or discharge), respectively. The Spearman correlation was used to check the association. Statistical significance is set at p < 0.05. CRP: C-reactive protein; CALLY index: CRP-albumin-lymphocyte index; SOFA: sequential organ failure assessment; SOFA_1: SOFA score at day 1; SOFA_diff: difference in SOFA score at day 7 from that at day 1.

**Figure 4:**
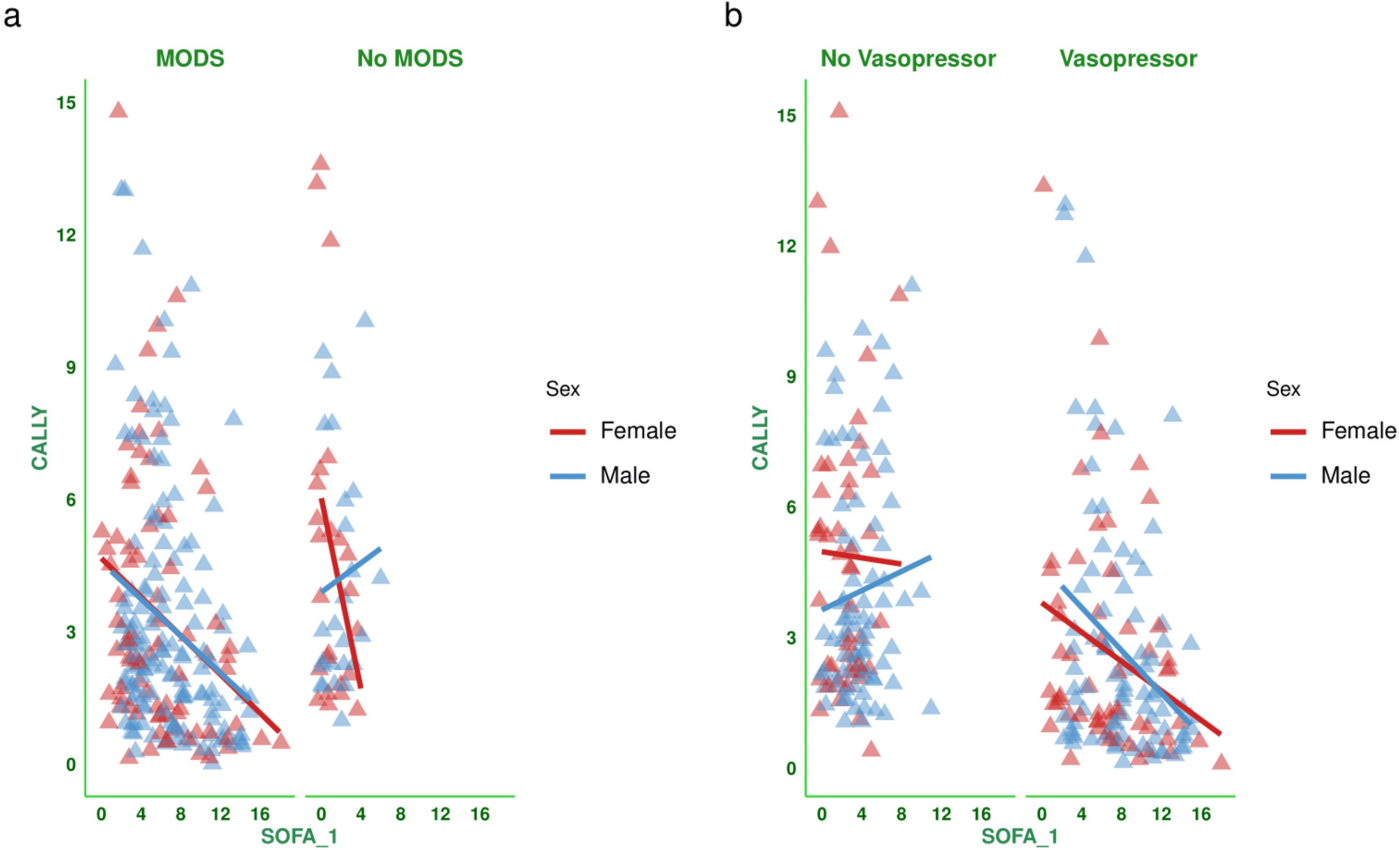
Correlation between CALLY index and SOFA score based on the presence of MODS and requirement of vasopressors The jitter plots show the correlation between the baseline SOFA score and the CALLY index in the study population. Panels (a and b) show the subgroup analyses based on the presence of MODS and the requirement for vasopressors, respectively. The Spearman correlation was used to check the association. Statistical significance is set at p < 0.05. CRP: C-reactive protein; CALLY index: CRP-albumin-lymphocyte index; SOFA: sequential organ failure assessment; SOFA_1: SOFA score at day 1; MODS: multiorgan dysfunction syndrome.

**Figure 5:**
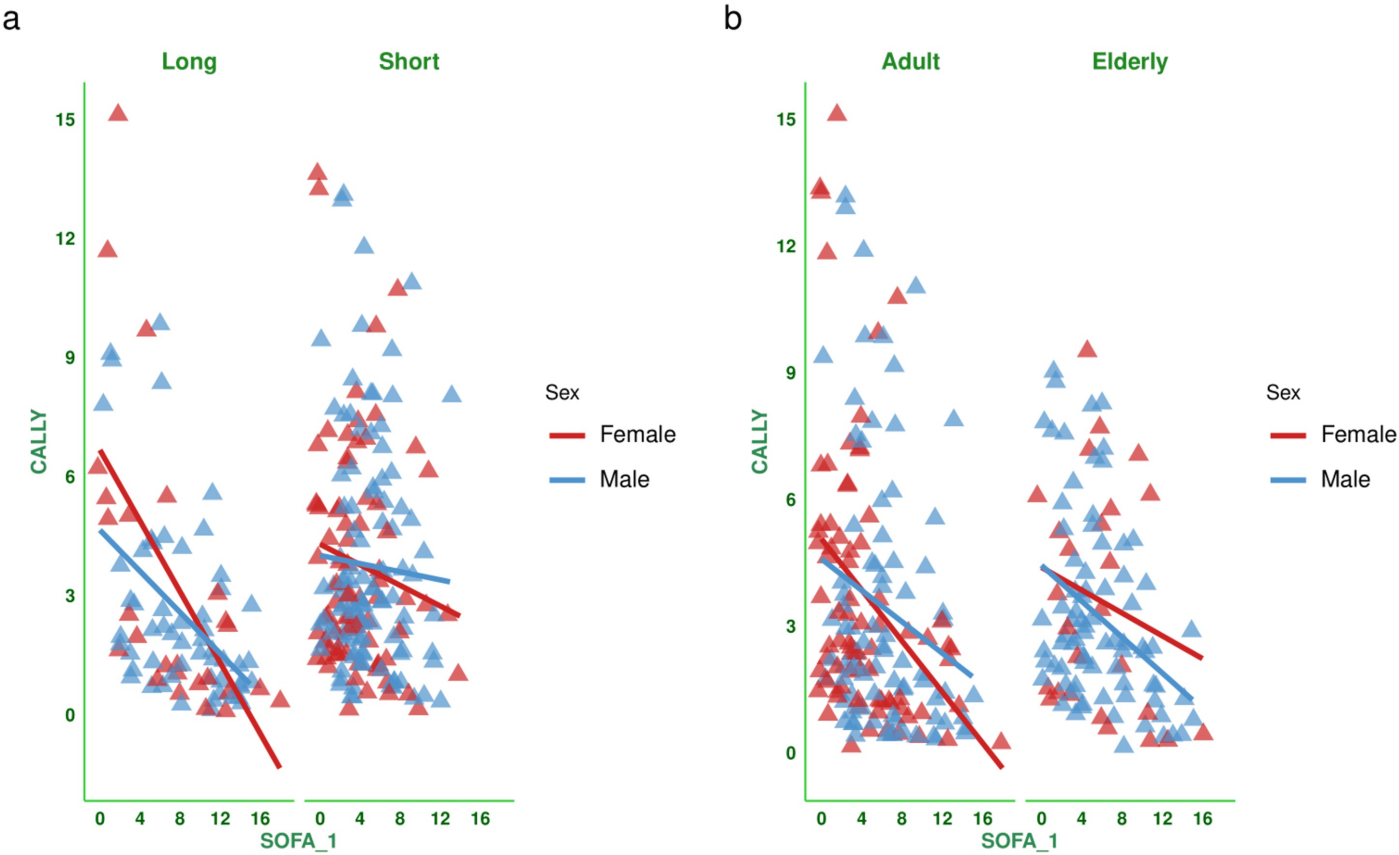
Correlation between CALLY index and SOFA score based on the duration of hospitalization and age group The jitter plots show the correlation between the baseline SOFA score and the CALLY index in the study population. Panels (a and b) show the subgroup analyses by hospitalization duration and age group, respectively. The Spearman correlation was used to check the association. Statistical significance is set at p < 0.05. CRP: C-reactive protein; CALLY index: CRP-albumin-lymphocyte index; SOFA: sequential organ failure assessment; SOFA_1: SOFA score at day 1.

Figure 2 shows the correlation between the baseline SOFA score and the CALLY index for the subjects. There was a negative correlation between the parameters (-0.299, 95% CI: -0.406 to -0.185, p < 0.001). Figure 3 shows the subgroup analysis of the above-mentioned parameter (i.e., the correlation between baseline SOFA score and CALLY index) based on the difference in SOFA score at day 7 (<2 or ≥2) and outcome (i.e., death or discharge). The correlations were weakly negative. Figure 4 shows the subgroup analysis of the correlation between baseline SOFA score and CALLY index, stratified by MODS (present or absent) and vasopressor (required or not). There were negative correlations between CALLY index and SOFA score (subjects with MODS: (-0.281, 95% CI: -0.400 to -0.153, p < 0.001), subjects without MODS: (-0.167, 95% CI: -0.428 to 0.119, p = 0.250), and subjects on vasopressors: (-0.297, 95% CI: -0.441 to -0.138, p < 0.001)). Figure 5 shows the subgroup analysis of the correlation between baseline SOFA score and CALLY index, stratified by hospitalization duration (>14 or ≤14 days) and age group (≤65 or >65 years). There were negative correlations between CALLY index and SOFA score (subjects with long hospital stay (i.e., >14 days): (-0.528, 95% CI: -0.669 to -0.350, p < 0.001), subjects with short hospital stay (i.e., ≤14 days): (-0.092, 95% CI: -0.234 to 0.054, p = 0.217), adult subjects (i.e., ≤65 years): (-0.294, 95% CI: -0.430 to -0.144, p < 0.001), and elderly subjects (i.e., >65 years): (-0.319, 95% CI: -0.481 to -0.135, p < 0.001)). Table 2 presents all correlation coefficients, their 95% CIs, and p-values.

**Table 2:** Correlation between CALLY index and SOFA score of the study population Spearman correlation was used to check the association. Statistical significance is set at p < 0.05. CRP: C-reactive protein; CALLY index: CRP-albumin-lymphocyte index; SOFA: sequential organ failure assessment; MODS: multiorgan dysfunction syndrome; CI: confidence interval.

Parameters	Total (n = 263)		Male (n = 161)		Female (n = 102)	
	r (95% CI)	p-value	r (95% CI)	p-value	r (95% CI)	p-value
Total	-0.299 (-0.406 to -0.185)	<0.001	-0.268 (-0.406 to -0.119)	<0.001	-0.333 (-0.495 to -0.148)	<0.001
SOFA_diff <2 and death	0.055 (-0.453 to 0.536)	0.840	-0.113 (-0.758 to 0.642)	0.788	0.159 (-0.615 to 0.777)	0.707
SOFA_diff ≥2 and death	-0.098 (-0.366 to 0.185)	0.498	-0.071 (-0.409 to 0.285)	0.701	-0.126 (-0.560 to 0.362)	0.619
SOFA_diff <2 and discharge	-0.054 (-0.205 to 0.100)	0.494	-0.008 (-0.203 to 0.188)	0.937	-0.095 (-0.335 to 0.157)	0.460
SOFA_diff ≥2 and discharge	-0.095 (-0.424 to 0.257)	0.600	-0.140 (-0.549 to 0.322)	0.555	-0.008 (-0.556 to 0.546)	0.981
MODS	-0.281 (-0.400 to -0.153)	<0.001	-0.269 (-0.419 to -0.106)	0.002	-0.298 (-0.488 to -0.080)	0.008
No MODS	-0.167 (-0.428 to 0.119)	0.250	0.095 (-0.312 to 0.472)	0.653	-0.394 (-0.688 to 0.012)	0.057
Vasopressor	-0.297 (-0.441 to -0.138)	<0.001	-0.321 (-0.500 to -0.116)	0.003	-0.278 (-0.504 to -0.016)	0.038
No vasopressor	0.022 (-0.157 to 0.199)	0.814	0.103 (-0.126 to 0.321)	0.377	-0.021 (-0.309 to 0.271)	0.891
Long duration	-0.528 (-0.669 to -0.350)	<0.001	-0.466 (-0.651 to -0.230)	<0.001	-0.604 (-0.803 to -0.283)	0.001
Short duration	-0.092 (-0.234 to 0.054)	0.217	-0.051 (-0.240 to 0.141)	0.601	-0.145 (-0.359 to 0.083)	0.211
Adult	-0.294 (-0.430 to -0.144)	<0.001	-0.213 (-0.411 to 0.004)	0.054	-0.371 (-0.551 to -0.159)	<0.001
Elderly	-0.319 (-0.481 to -0.135)	<0.001	-0.370 (-0.546 to -0.162)	<0.001	-0.206 (-0.550 to 0.197)	0.312

## Discussion

In this observational study, 263 patients were assessed. Their median age was 62.0 (52.0-73.0) years. Men comprised 61.22% of the study population. The median duration of hospitalization was 12.0 (9.0-16.0) days. The median CRP, serum albumin, and lymphocyte count values of the study population were 112.97 (77.95-206.82) mg/L, 2.78 (2.36-3.17) g/dL, and 1.09 (0.67-1.72) x 10^9^/L. The median CALLY index of the subjects was 2.48 (1.34-4.94). The median SOFA score during admission was 5.0 (3.0-8.0). There was a negative correlation between SOFA score at day 1 and CALLY index (-0.299, 95% CI: -0.406 to -0.185, p < 0.001). Male and female subjects also showed similar negative correlations. The subgroup analyses also demonstrated similar findings. Our observations regarding the CALLY index matched those of Zhang et al. [15] and Altuntaş et al. [20].

SOFA scores are widely used to evaluate the morbidity and prognosis of critically ill patients. However, its computation demands time and multiple variables [7-9]. CRP, procalcitonin, adrenomedullin, IL-6, and ICAM-1 are recent biomarkers for sepsis [10,11]. The systemic immune-inflammation index (SII) is another indicator of immune-mediated inflammatory conditions and sepsis [21,22]. Nowadays, the CALLY index has emerged as an efficient composite biomarker for sepsis, encompassing nutritional aspects alongside immune and inflammatory responses [14-16]. Lower serum albumin levels are generally observed in malnourished individuals [23]. Our body’s immune function is positively correlated with the lymphocyte counts [24]. Increased CRP levels are linked with muscle wasting, catabolic processes, and gradual functional decline in elderly individuals [23,24]. Therefore, these three parameters strengthen the CALLY index by connecting nutritional status with immune function and systemic inflammation [23].

Strength and limitation of the study

The evaluation and correlation of the CALLY index and the SOFA score among the study subjects strengthened our study. Our study had a few limitations. First, the observational study design and a lower sample size hinder further analysis of the indices. Second, missing data on antibiotics used; hemodynamic, renal, hepatic, and glycemic parameters; comorbidities; concomitant medications; daily fluid intake; and urine output prompted us to exclude some patients. Third, referred patients were excluded because their indices could have been miscalculated owing to previous interventions.

## Conclusions

The study population had elevated CRP and lower serum albumin values. Male and female participants had similar serum albumin, CRP, lymphocyte count, CALLY indices, and SOFA scores. The correlation between the CALLY index and the SOFA score was weakly negative. The subgroup analyses demonstrated similar data. Our study findings cannot be generalized due to a single-center observational study design, a small sample size, and missing data on antibiotics used; renal, hepatic, glycemic, and hemodynamic parameters; concomitant medications; comorbidities; daily fluid intake; and urine output. We suggest prospective studies with larger sample sizes and longer follow-up to evaluate the potential of the CALLY index as a biomarker for sepsis.

## References

[1] Jain A, Singam A, Mudiganti VN (2024). Recent advances in immunomodulatory therapy in sepsis: a comprehensive review. Cureus.

[2] Zhang X, Zhang Y, Yuan S, Zhang J (2024). The potential immunological mechanisms of sepsis. Front Immunol.

[3] Konjety P, Chakole VG (2024). Beyond the horizon: a comprehensive review of contemporary strategies in sepsis management encompassing predictors, diagnostic tools, and therapeutic advances. Cureus.

[4] Di Raimondo D, Pirera E, Rizzo G, Simonetta I, Musiari G, Tuttolomondo A (2022). Non-coding RNA networks as potential novel biomarker and therapeutic target for sepsis and sepsis-related multi-organ failure. Diagnostics (Basel).

[5] Baig MM, GholamHosseini H, Afifi S, Lindén M (2021). A systematic review of rapid response applications based on early warning score for early detection of inpatient deterioration. Inform Health Soc Care.

[6] Gerry S, Bonnici T, Birks J, Kirtley S, Virdee PS, Watkinson PJ, Collins GS (2020). Early warning scores for detecting deterioration in adult hospital patients: systematic review and critical appraisal of methodology. BMJ.

[7] Vincent JL, Moreno R, Takala J (1996). The SOFA (Sepsis-related Organ Failure Assessment) score to describe organ dysfunction/failure. On behalf of the Working Group on Sepsis-Related Problems of the European Society of Intensive Care Medicine. Intensive Care Med.

[8] Moreno R, Rhodes A, Piquilloud L (2023). The Sequential Organ Failure Assessment (SOFA) score: has the time come for an update?. Crit Care.

[9] Koch C, Edinger F, Fischer T (2020). Comparison of qSOFA score, SOFA score, and SIRS criteria for the prediction of infection and mortality among surgical intermediate and intensive care patients. World J Emerg Surg.

[10] Ahuja N, Mishra A, Gupta R, Ray S (2023). Biomarkers in sepsis-looking for the Holy Grail or chasing a mirage!. World J Crit Care Med.

[11] Barichello T, Generoso JS, Singer M, Dal-Pizzol F (2022). Biomarkers for sepsis: more than just fever and leukocytosis-a narrative review. Crit Care.

[12] He RR, Yue GL, Dong ML, Wang JQ, Cheng C (2024). Sepsis biomarkers: advancements and clinical applications-a narrative review. Int J Mol Sci.

[13] Müller L, Hahn F, Mähringer-Kunz A (2021). Immunonutritive scoring for patients with hepatocellular carcinoma undergoing transarterial chemoembolization: evaluation of the CALLY index. Cancers (Basel).

[14] Li Y, Wei Q, Ke X (2024). Higher CALLY index levels indicate lower sarcopenia risk among middle-aged and elderly community residents as well as hospitalized patients. Sci Rep.

[15] Zhang Y, Yang Z, Feng Y (2025). Development and validation of nomogram models incorporating the inflammatory nutritional index CALLY for predicting survival in locally advanced rectal cancer after neoadjuvant chemoradiotherapy. Cancer Manag Res.

[16] Geng M, Zhang K (2025). CRP-albumin-lymphocyte index (CALLYI) as a risk-predicting biomarker in association with osteoarthritis. Arthritis Res Ther.

[17] Seymour CW, Liu VX, Iwashyna TJ (2016). Assessment of clinical criteria for sepsis: for the third international consensus definitions for sepsis and septic shock (Sepsis-3). JAMA.

[18] Khairnar MR, Wadgave U, Shimpi PV (2017). Kuppuswamy's socio-economic status scale: a revision of occupation and income criteria for 2016. Indian J Pediatr.

[19] (2026). R: a language and environment for statistical computing, Vienna, Austria. https://www.r-project.org/.

[20] Altuntaş G, Yıldırım R, Demirel İ (2025). Superiority of pan-immune inflammation value, systemic inflammation index, and CALLY scores prognostic value for mortality of ischemic stroke patients followed in intensive care unit. BMC Immunol.

[21] Patro S, Sharma V, Choudhary A, Varuneil Y, Arora P, Nayak S, Sahoo JP (2025). Systemic immune-inflammation index (SII) of patients with and without diabetic neuropathy: a cross-sectional study. Cureus.

[22] Mangalesh S, Dudani S, Malik A (2023). The systemic immune-inflammation index in predicting sepsis mortality. Postgrad Med.

[23] Mancinetti F, Guazzarini AG, Gaspari M, Croce MF, Serra R, Mecocci P, Boccardi V (2025). Integrating nutrition, inflammation, and immunity: the CALLY index as a novel prognostic biomarker in acute geriatric care. Nutrients.

[24] Xu L, Li C, Aiello AE (2025). Compositional analysis of lymphocytes and their relationship with health outcomes: findings from the health and retirement study. Immun Ageing.

